# Quality of life in bladder cancer patients receiving medical oncological treatment; a systematic review of the literature

**DOI:** 10.1186/s12955-018-1077-6

**Published:** 2019-01-22

**Authors:** G. A. Taarnhøj, C. Johansen, H. Pappot

**Affiliations:** 1 Department of Oncology, Rigshospitalet, Blegdamsvej 9, section 5073, 2100 Copenhagen Ø, Denmark; 2 Unit of Survivorship, Danish Cancer Society, Strandboulevarden 49, 2100 Copenhagen Ø, Denmark

**Keywords:** Bladder cancer, Chemotherapy, Muscle-invasive bladder cancer, Quality of life, Urothelial cancer

## Abstract

**Background:**

Previous quality of life (QoL) literature in bladder cancer (BC) patients has focused on finding the preferred urinary diversion while little is known about the QoL of patients in medical oncological treatment (MOT). We performed a systematic review to assess the existing literature on QoL in patients with muscle-invasive BC (MIBC) undergoing MOT.

**Methods:**

A systematic search of Pubmed and Embase was performed. Inclusion criteria were studies containing QoL data for patients undergoing chemo- and/or radiotherapy. We extracted all QoL scorings at different time intervals and on the six most prevalent domains: overall QoL, urinary, bowel sexual symptoms, pain and fatigue. The study was carried out according to PRISMA guidelines for systematic reviews and GRADE was used to rate the quality of evidence from the included studies.

**Results:**

Of 208 papers reviewed, 21 papers were included. Twenty-one different QoL instruments were applied. The only data on QoL during chemotherapy was from patients in clinical trials investigating new treatments. No studies were found for patients in neoadjuvant treatment. The level of evidence at each time point was graded as very low to moderate. From the studies included the overall QoL seemed inversely related to the organ-specific impairment from sexual and urinary symptoms and increased with decreasing organ-specific symptoms for long term survivors > 6 months after treatment.

**Conclusions:**

Collection of data on QoL from patients with MIBC disease undergoing MOT has been sparse and diverse. The present data can act as a summary but prompts for more prospective collection of QoL data from BC patients.

**Electronic supplementary material:**

The online version of this article (10.1186/s12955-018-1077-6) contains supplementary material, which is available to authorized users.

## Background

Despite recent literature highlighting the evident benefit of regular symptom reporting and early handling of side effects by patient-reported outcomes, the implementation of such in daily practice has yet to occur [1–3]. For patients receiving chemotherapy, clinical trials have traditionally informed us about quality of life (QoL) for these patients retrospectively as part of study reporting. These reports, however, inform us about highly selected patient populations eligible for enrollment in clinical trials, and are often not containing cancer specific modules as highlighted by three reviews in the field of bladder cancer published 1999–2005 [4–6]. Thus, our knowledge of QoL outside clinical trials remains sparse.

Bladder cancer (BC) patients are characterized by heterogenic prognostics due to variation in their extent of disease as illustrated by the division into non-muscle invasive (NMIBC)/TaT1CIS, muscle invasive bladder cancer (MIBC)/T2-T4 and metastatic BC. Great interest and effort has been put into understanding health-related quality of life and symptomatic issues affecting the overall QoL for MIBC patients having undergone surgical procedures [7, 8]. Little is to the authors’ knowledge known of the BC patients undergoing medical oncological treatment.

As part of the planning of a randomized patient-reported outcomes study in the BC population receiving medical oncological treatment, we set out to review the current literature for BC patients receiving chemotherapy. The aim of this study is to gather evidence of the QoL issues affecting the lives of BC patients during all phases of their disease, from diagnosis to treatment and thereafter thus informing us of potential gaps in the literature. The results will furthermore assist in determining which symptomatic patient-reported outcomes to be used in a coming randomized trial. We therefore present a systematic review of the QoL literature published on patients with locally advanced or metastatic BC undergoing chemotherapy.

## Methods

### Search criteria

A systematic search was performed in PubMed using the MeSH-terms ‘quality of life’, ‘urinary bladder neoplasms’, ‘drug therapy’ and included ‘quality of life’, ‘bladder cancer’ and ‘chemotherapy’ as title or abstract terms (Additional file 1). The same search strategy was used in Embase. A professional, full-time librarian assisted the search to ensure systematics. The results were examined by title (author GAT) and if found relevant abstract and papers were read (GAT). Papers were found relevant if they included quantitative QoL data from patients with MIBC undergoing chemotherapy at any time point before, during or after their diagnosis or treatment. Radiotherapy as treatment modality was included because no papers with post-treatment QoL data were found for patients having undergone chemotherapy. To limit the search to the relevant population the following exclusion criteria were applied:

- not available in English
- published before 2000 thereby allowing for a slight overlap with the previous reviews in order not to dismiss valuable studies
- only comparing surgical procedures
- only involving NMIBC
- only abstract available.

**Fig. 1:**
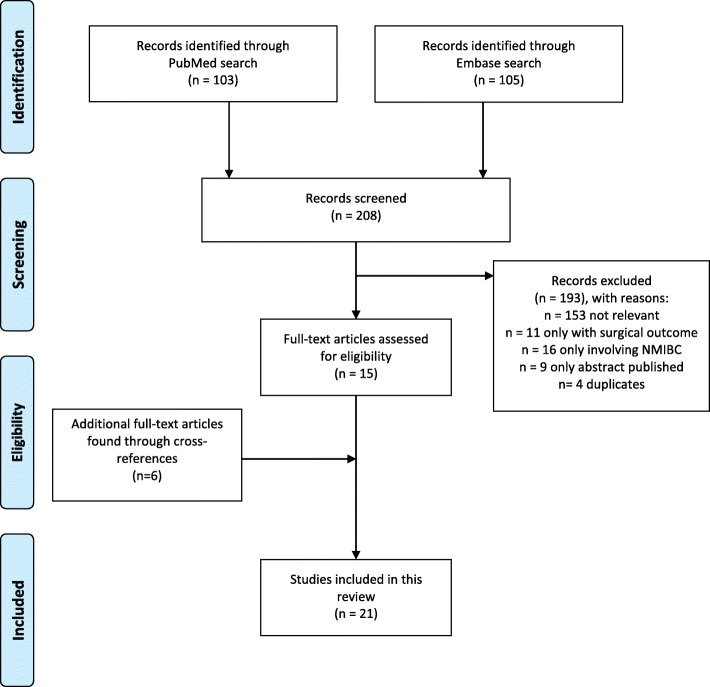
Flow chart in PRISMA consort diagram of the screening and selection of studies

Finally, to expand the results due to a modest number of relevant studies, cross-references in the included articles were examined. The search is graphically presented according to the PRISMA consort diagram (Fig. 1) and was carried out according to the PRISMA guidelines as a systematic review (Additional file 2) [9].

### Data extraction and analysis

**Fig. 2:**
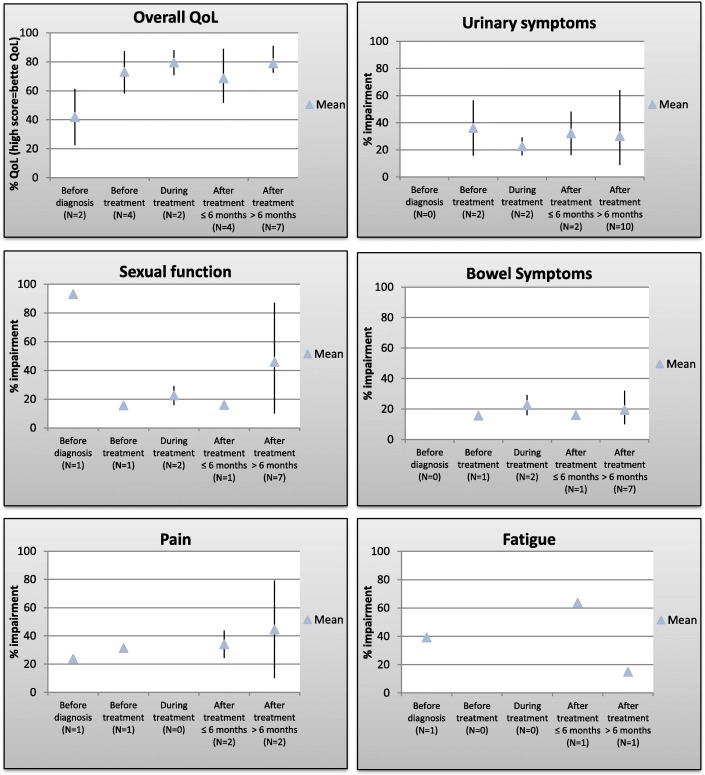
Summarized quality of life scores during disease phases for bladder cancer patients

In order to construct graphs eliciting QoL over time, all QoL measurements from the included papers (Table 1) were noted. All scores on other scales than 0–100 were converted to a 0–100 scale. Overall QoL is displayed with values 0–100, with increasing scores illustrating a better QoL, while the domain specific items are presented with a 0–100 scale, with an increasing score implicating more impairment, displayed in Table 2 as percentage impairment. The questionnaires with a reverse scaling of the domain specific items than described above (the FACT-BL, the EPIC and the domain-specific part of the SF-12), are scored on a Likert-like scale with increasing scores representing better QoL/fewer symptoms. These scorings were reverse transformed to represent percentage impairment for comparability with the other studies. The FACT-BL questionnaire scores comprise urinary, bowel and sexual symptom distress in one score, thereby disabling unique symptom scores for these items. If no subgrouping into urinary, sexual and bowel scores was specified for the studies applying the FACT-BL, the FACT-BL scores were thus used in Fig. 2 for the graphical presentation of urinary, bowel and sexual symptoms, even though the FACT-BL score then was used multiple times, thereby perhaps over- or underestimating the given symptom. No scores from patients having undergone primary surgical treatment (radical cystectomy) were included in the analysis, thereby allowing summarized scores without the known post-operative and instrumental issues known to affect QoL influencing the results of this review.

**Table 1:** Published studies concerning bladder cancer and quality of life 2000–2018, *N* = 21

Topic	Authors	Year	Patient group	QoL instrument	Nb. of patients	Outcome	Main symptom topics	Limitations
QoL before cystoscopy	Goossens-Laan et al. [12]	2013	All pts. with hematuria before cystoscopy: BC pts. vs. pts. with hematuria of other causes (OC)	WHOQOL-BREF, SF-12, IIEF, STAI-10	476 (87 BC pts.(61 NMIBC, 26 MIBC), 389 OC)	QoL comparable between BC and OC groups, Erectile dysfunction highest in BC group, MIBC lowest percentage of anxious personalities of pts. with BC.	Erectile dysfunction Anxiety General health perceptions, physical, emotional, social, fatigue, pain	Selected population (hematuria), only 26 MIBC pts. IIEF not used consistently
QoL before and after RT and concurrent CT	Lagrange et al. [27]	2011	BC T2a-T4 before and up to 3 years after TUR + concurrent RT/CT (Phase II)	QLQ-C30 + BC specific questions + LENT-SOMA	51 RT/CT	Satisfactory QoL for 67% of pts. Decrease in BC specific symptoms over time.	Bladder function Global health score Physical, personal, emotional, cognitive, social functioning	No data during treatment, QoL limited to pts. alive without disease
	Fung et al. [11]	2014	BC before and after BC diagnosis and treatment (77–88% NMIBC)	SF-36/VR-12	1476 (620 before, 856 after)	Deteriorating physical & mental component scores pre and post diagnosis. MIBC also clinically significant that persisted up to 10 yrs. after diagnosis. Co-morbidity a risk factor.	Physical and mental scores	Cross-sectional study, not comparable, only 179 BC pts. with 2 questionnaires. No data on treatment.
QoL during treatment (TUR/RT/CT)	Albers et al. [19]	2002	Gemcitabine for platinum-resistant or metastatic BC pts. (Phase II)	Validated QoL questionnaire (Spitzer et al.)	25 CT	No overall difference during treatment, pain decreased significantly amongst responders, pain increased overall	Overall QoL, pain	Questionnaire not comparable to others.
	Roychowdhury et al. [14]	2003	GCis vs MVAC in metastatic BC pts. (Phase III)	EORTC QLQ-C30	326 (165 GCis, 161 MVAC)	Equal QoL between the two groups. Improvement in fatigue during treatment, not significant.	Overall QoL, fatigue	No BC specific questions, only metastatic disease, only baseline values
	Herman et al. [18]	2004	Concurrent gemcitabine +RT, BC T2-T3 (Phase I)	FACT-G + FACT-BL	23 RT/CT	Treatment related QoL: no significant differences were found	Urinary, bowel, erectile, global QoL	Few pts., no follow-up post treatment
	Butt et al. [37]	2008	Advanced cancer in chemotherapy (many cancers, stages 3–4)	FACT-G + FACT-BL + non-validated instruments	31 CT	Identified 5 major symptoms/concerns	Fatigue (48.4%), anxiety about progression, family worries, enjoy life, control bowels	No baseline data, no specification on when pts. answered questionnaire. No specific numbers for BC pts.
	Joly et al. [16]	2009	Weekly paclitaxel for recurrence (Phase II), 93% metastatic BC pts.	FACT-G + FACT-BL + FACT-Taxane	45 CT	No decrease in QoL scores, few patients experiences improvement on one or more parameters.	Overall QoL	Limited number of patients. QoL for non-responders not displayed.
	De Santis et al. [17]	2012	GCar vs. M-CAVI in BC T3–4 w/N+/M+ disease, not fit for cystectomy or cisplatin (Phase II/III)	EORTC QLQ-C30	238 (119 GCar, 119 M-CAVI)	No difference in QoL between the two arms	Overall	50% response rate after baseline. No QoL data provided
	Huddart et al. [22]	2017	Selective bladder preservation vs. RC	EORTC QLQ-C30 + QLQ-BLM30	20 SBP	Stable QoL during and after treatment for SBP group	Overall, bowel, sexual	Baseline changes reported, no quantitative data.
QoL after treatment (TUR/RT/CT/RC)	Henningsohn et al. [23]	2002	All pts. MIBC, stages not specified, 1–19 yrs. after RT vs after cystectomy	Non-validated, self-made	309 (58 RT, 251 cystectomy) + controls	Small diff. in sexual disturbances btw. The two groups.	Bowel, urinary, sexual, well-being (energy, anxiety, depression), lymphoedema	Older RT group, no adjustment.
	Zietman et al. [24]	2003	BC T2-4a, disease free, after TUR + RT + CT (+ salvage cystectomy), 1–15 yrs. after treatment.	Non-validated BC and RT specific questionnaires + SF-36	49 (31 with total completion)	Urinary functions like general population, + bowel problems	Urinary, bowel, sexual, global HRQOL	No comparison (baseline or other).
	Matsuda et al. [35]	2003	BC Ta-T1:80%, T2-T4 20%), 5 yrs. after treatment	FACT-G+ FACT-BL	95 (no subgrouping specified)	Poor sexual function after cystectomy	Overall QoL, social, functional, physical emotional well-being, urinary	Primarily (80%) Ta + T1 tumors, 4 pts. received CT. Only > 5 yr. survivors.
	Fokdal et al. [26]	2004	BC T1-4bNxM0, 3–10 yrs. after RT treatment	LENT-SOMA	53 RT, 63 population controls	Significantly more bothersome urinary, bowel and sexual symptoms in patients vs. controls.	Urinary, bowel, sexual	Retrospective, no baseline data. Telephone questionnaire by study leader, all disease free.
	Allareddy et al. [25]	2006	BC Tis-T4, 6–15 yrs. after RC vs. IB treatment	FACT-G + FACT-BL	82 RC, 177 IB	Poor sexual function after RC, no difference in overall scores	Overall QoL, sexual, urinary, bowel	
	Hashine et al. [21]	2008	MIBC pts., but stages not specified, 1–14 yrs. after TUR + RT + CT vs TUR-BT for NMIBC	IPSS, SF-36 + EPIC	33 MIBC vs 128 NMIBC	Comparable QoL in the two groups	Overall QoL, physical functioning, role-physical, urinary, bowel, pain.	Retrospective, no baseline data, NMIBC as controls
	Singer et el. [30]	2013	BC, 64% MIBC and 26% NMIBC vs general population, 9 days-37 yrs. after treatment.	EORTC QLQ-C30	823 (210 NMIBC, 530 MIBC, 83 unknown), 2037 general population	Significantly worse QoL for BC vs general population, CT and RT + CT associated with more dyspnea, appetite loss, social functioning, constipation, nausea & vomiting	Physical, emotional, social, role, cognitive, global HS, fatigue, nausea, pain, dyspnea, insomnia, appetite loss, constipation, diarrhea, financial difficulties	Selection bias (inpatient, BC population not representative and not adjusted for comorbidities or extent of disease, different follow-up periods)
	Kent et al. [38]	2014	26% BC, 2–5 yrs. post disease	SF-36	161(no subgrouping specified)	Pts with low income, Hispanic ethnicity and pts. with recurrence experienced more bothersome symptoms.	24% with symptoms. Of these: urinary symptoms, procedural pain, fatigue, diarrhea, abd. Pain, neuropathy, pain, rash.	No baseline data.
	Mak et al. [20]	2016	MIBC T2-T4, 2–16 yrs. after RC vs TMT (TURB+CT + RT)	EuroQOL EQ-5D, EORTC QLQ-C30, QLQ-BLM30, EPIC, CTPS, IOCv2	173 (109 RC, 64 TMT)	Good Qol for RC &TMT, TMT had better bowel function and better sexual function	Overall QoL, urinary, bowel, sexual,	No baseline data, all patients > 2 yrs. free of disease,not prospective, different follow-up times
	Mason et al. [36]	2018	MIBC + NMIBC, unknown disease stage	EQ-5D-5 L + FACT-BL + SDI-21	34 CT/RT + 61 RT	RT/CT group with more fatigue, more social distress, more anxiety but more content with sexual life than RC group	Overall QoL, mobility, self-care, pain, anxiety, social distress, financial, fatigue, sexual, bowel, urinary, emotional, functional well-being	Only prevalence of problems, no comparable QoL scores
	Perlis et al. [13]	2018	MIBC post CT	BUSS	34 CT + 15 CT/RT	Lower QoL compared to non-CT receivers, especially for patients with metastatic disease	Overall QoL, anxiety, fatigue, pain, urinary, bowel, sexual impairment	Validation study, only QoL scores for overall QoL, no specific time point, estimated at least 1 year free of disease

Abbreviations: *QoL* quality of life, *BC* bladder cancer, *MIBC* muscle invasive bladder cancer, *NMIBC* non-muscle invasive bladder cancer. *RC* radical cystectomy, *RT* radiotherapy, *CT* chemotherapy, *SBP* selective bladder preservation, *IB* intact bladder, *TUR* transurethral resection, *TMT* trimodality treatment, *BT* intravesical Bacillus Calmette-Guerin therapy, *GCi* gemcitabine/cisplatin, *GCar* gemcitabine/carboplatin, *MVAC*:methotrexate/vinblastine/doxorubicin/cisplatin, *M-CAVI*: methotrexate/carboplatin/vinblastine. See Table 7 for abbreviations of QoL instruments

**Table 2:** Quality of life scores from the included studies

Authors	QoL results	Time point		Value	Reference interval	Percent impairment (overall QoL on opposite scaling)
Goossens-Laan et al. [12]	WHOQOL-bref: overall QoL	before diagnosis		3.8	2-10	22.5
	SF-12 overall (sum of general domains /6)				0-100	61.4
	SF-12: bodily pain			76.2	0-100	23.8
	SF-12: fatigue			60.7	0-100	39.3
	IIEF: erectile dysfunction			93.1	0-100	93.1
Lagrange et al. [27]	QLQ-C30: Overall QoL	before treatment		68	0-100	68
		after treatment	6 mths	60	0-100	60
			12 mths	81	0-100	81
			24 mths	68	0-100	68
			36 mths	71	0-100	71
	Non-validated: urinary	before treatment		56.5	0-100	56.5
		after treatment	6 mths	48.2	0-100	48.2
			12 mths	30	0-100	30
			24 mths	37.5	0-100	37.5
			36 mths	64.3	0-100	64.3
Fung et al. [11]	Scores not in comparable scale					
Roychowdhury et al. [14]	QLQ-C30: Overall QoL	Baseline before CT		58.3	0-100	58.3
	Pain	Baseline before CT		33.3	0-100	33.3
	Fatigue	Baseline before CT		33.3	0-100	33.3
Albers et al. [19]	Spitzer: overall QoL	before CT (after TUR)		7.9	0-10	79
		after CT	at end of treatment	7.4	0-10	74
	Spitzer: pain	before CT (after TUR)		4.8	0-7	52
		after CT	at end of treatment	5.3	0-7	47
Herman et al. [18]	FACT-G: overall	before		94.5	0-108	87.5
		during	3 weeks after initiating treatment	95.2	0-108	88.1
		after	2 weeks after treatment(8 wks after treatment initiation)	96.2	0-108	89
	FACT-BL	before		37.1	0-156	76.2
		during	3 weeks after initiating treatment	36	0-156	76.9
		after	2 weeks after treatment(8 wks after treatment initiation)	34.3	0-156	78
	subcategory: bowel	before, during, after				71
	subcategory: erectile function	before, during, after				58
Butt et al. [37]	Overview paper, not specified for BC patients					
Joly et al. [16]	FACT-G: overall	during	1 week after start of treatment	77	0-108	71.3
			2 weeks	79	0-108	73.1
			3 weeks	82	0-108	75.9
			4 weeks	78	0-108	72.2
			5 weeks	72	0-108	66.7
			6 weeks	71	0-108	65.7
	FACT-BL	during	1 week after start of treatment	107	0-156	31.4
			2 weeks	110	0-156	29.5
			3 weeks	117	0-156	25
			4 weeks	118	0-156	24.4
			5 weeks	104	0-156	33.3
			6 weeks	104	0-156	33.3
De Santis et al. [17]	No QoL scores listed					
Huddart et el. [22]	Scores not in comparable scale					
Henningsohn et al. [23]	Non-validated: urinary	after treatment	1-19 yrs after RT treatment	41	0-100	41
	Non-validated: bowel	after treatment	1-19 yrs after RT treatment	22.75	0-100	22.75
	Non-validated: sexual	after treatment	1-19 yrs after RT treatment	62	0-100	62
Zietman et al. [24]	Non-validated: urinary	after treatment	1-15 yrs after treatment	13.3	0-100	13.3
	Non-validated: bowel	after treatment	1-15 yrs after treatment	14.3	0-100	14.3
	Prostate instrument/Index for women: sexual	after treatment	1-15 yrs after treatment	53.7	0-100	53.7
	SF-36: overall	after treatment	1-15 yrs after treatment	74	0-100	74
Matsuda et al. [35]	FACT-G: overall	after	5-10 yrs	81	0-108	75
	FACT-BL	after	5-10 yrs	116.8	0-156	25.1
Fokdal et al. [26]	LENTSOMA: urinary	after	3-10 yrs after treatment	42.3	0-100	42.3
	bowel	after	3-10 yrs after treatment	32	0-100	32
	sexual	after	3-10 yrs after treatment	89	0-100	89
Allareddy et al. [25]	FACT-G: overall	after	6-16 yrs	89	0-104	85.6
	FACT-BL	after	6-16 yrs	125	0-156	17.8
	sexual function	after	6-16 yrs	32	0-100	32
Hashine et al. [21]	SF-36: overall (sum of all scales /8)	after	1-14 yrs after RT treatment	73	0-100	73
	subcategory: body pain	after	1-14 yrs after RT treatment	79.2	0-100	79.2
	EPIC: bowel	after	1-14 yrs after RT treatment	90	0-100	90
	EPIC: urinary	after	1-14 yrs after RT treatment	92	0-100	92
	EPIC: sexual	after	1-14 yrs after RT treatment	90	0-100	90
Singer et el. [34]	QLQ-C30: overall (sum of general scales /6)	after	0-37 yrs after	51.7	0-100	51.7
	Subcategory: pain	after	0-37 yrs after	43.8	0-100	43.8
	Subcategory: fatigue	after	0-37 yrs after	63.7	0-100	63.7
Kent et al. [38]	Overview paper, not specified for BC patients					
Mak et. al. [20]	QLQ-C30: Overall (sum of general scales /6)	after	2-16 yrs after treatment	88.2	0-100	88.2
	Subcategory: fatigue			15	0-100	15
	Subcategory: pain			10	0-100	10
	EQ-5D(3L): overall			91	0-100	91
	EQ-5D:VAS: overall			81	0-100	81
	QLQ-BLM30: urinary			22	0-100	22
	QLQ-BLM30: sexual			52	0-100	52
	EPIC: bowel			87	0-100	87
Mason et al. [36]	EQ5D		1-5 years post diagnosis, no comparable scores.			
Perlis et al. [13]	BUSS: overall QoL	After	>1 year after	72.5	0-100	72.5

Abbreviations: QoL: quality of life, BC: bladder cancer, RT: radiotherapy, CT: chemotherapy, TUR: transurethral resection. See table 4 for abbreviations of QoL instruments

The GRADE criteria were applied for systematic review of the studies included [10]. GRADE is a system for assessing the quality of the evidence for the chosen outcome. The chosen outcome in this review was the determination of whether the sum of the studies included at each time point could act as reliable evidence for assessing QoL for the given population. Downgrading was considered based on either risk of bias, inconsistency, indirectness, imprecision or apparent publication bias. A risk of bias was assessed if the studies included e.g. reported different outcomes, if the outcomes from one study were diverging or low compliance in the study introduced selection bias. Inconsistency was determined if there was a large amount of clinical heterogeneity across the studies, participants or outcomes or if the methods applied were different across studies. Indirectness was determined if the population or outcomes differed from the population or outcomes of interest. Imprecision was established if the number of patients included in the rating of the outcome was too small to give a valid estimate of the outcomes. Publication bias was assessed by funnel plots looking at the pattern of study results [10]. Reasons for each downgrading assessment according to the categories above along with the findings of this analysis are given in Tables 3, 4, 5, 6 and the assessments reflect the degree of confidence we can have of the summarized QoL scores described above. The GRADE evaluation was done by two authors (GAT, HP).

**Table 3:** List of quality of life instruments applied, *N*=18

Abbreviated QoL instrument	Full Title
EORTC QLQ-C30	European Organization for Research and Treatment of Cancer Quality of life Core Questionnaire – for all cancer patients
EORTC QLQ-BLM30	European Organization for Research and Treatment of Cancer Quality of life module for muscle invasive bladder cancer
LENT-SOMA	Late Effects in Normal Tissue – Subjective, Objective, Management and Analytic scale for late effects of radiotherapy
SF-36	RAND Medical Outcomes Study Short Form 36
SF-12	RAND Medical Outcomes Study Short Form-12
EPIC	Expanded Prostate Cancer Index Composite
FACT-G	Functional Assessment of Cancer Therapy – General – for all cancer patients
FACT-BL	Functional Assessment of Cancer Therapy – for patients with bladder cancer
FACT-Taxane	Functional Assessment of Cancer Therapy – for patients receiving taxane therapy
IPSS	International Prostate Symptom Score
EuroQOL EQ-5D	EuroQOL Group non-disease specific QoL instrument
CTPS	Cancer Treatment Perception Scale
IOCv2	Impact of Cancer version 2
HADS	Hospital Anxiety and Depression Scale
WHOQOL-BREF	World Health Organization Quality of Life abbreviated version
STAI-10	State-Trait Anxiety Inventory-Trait scale short form
IIEF	International Index of Erectile Function
Spitzer index	Validated instrument for palliative patients

**Table 4:** GRADE Summary of findings. Outcome: Urinary symptoms

	GRADE issues					Overall GRADE rating	
	Nb. of studies	Risk of bias	Inconsistency	Indirectness	Imprecision	Publication bias	
Before diagnosis	2 [18, 27]		X^1^	X^2^	X^3^		
Before treatment	0	–	–	–	–	–	N/A
During treatment	2 [16, 18]		X^4^	X^5^	X^3^		
≤ 6 months after treatment	2 [18, 27]		X^1^		X^3^		
> 6 months after treatment	8 [20, 21, 23–27, 35]	X^6^, X^7^	X^8^	X^9^		None apparent^10^	

^1^1 out of 2 scores from non-validated questionnaire [27]

^2^No studies before CT for metastatic disease or as neoadjuvant treatment

^3^Large difference in populations hence diverging scores

^4^Small number of patients

^5^Only one study representing metastatic population, no studies representing neoadjuvant population

^6^Diverging scores from one study [27]

^7^Small number of patients for [27] at time point 36 months

^8^Different incl. Non-validated questionnaires used

^9^All patients after CT/RT or RT alone

^10^As estimated by forest plot

**Table 5:** GRADE Summary of findings. Outcome: Sexual impairment

		GRADE issues					Overall GRADE rating
	Nb. of studies	Risk of bias	Inconsistency	Indirectness	Imprecision	Publication bias	
Before diagnosis	1 [12]	X^1^, X^2^			X^3^		
Before treatment	1 [18]	X^1^			X^3^		
During treatment	2 [16, 18]		X^4^	X^5^	X^3^		
≤ 6 months after treatment	1 [18]	X^1^			X^3^		
> 6 months after treatment	7 [20, 21, 23–26, 35]		X^6^			None apparent^7^	

^1^Only one study represented

^2^Selected population

^3^Small number of patients

^4^Large difference in populations hence diverging scores, despite same instrument used

^5^Only one study representing metastatic population, no studies representing neoadjuvant population

^6^Large range between scores despite comparable study populations, presumably due to different instruments used, incl. Non-validated instrument

^7^As estimated by funnel plo

v

**Table 6:** GRADE Summary of findings. Outcome: Bowel symptoms

		GRADE issues					Overall GRADE rating
	Nb. of studies	Risk of bias	Inconsistency	Indirectness	Imprecision	Publication bias	
Before diagnosis	0	–	–	–	–	–	N/A
Before treatment	1 [18]	X^1^			X^2^		
During treatment	2 [16, 18]		X^3^	X^4^	X^2^		
≤ 6 months after treatment	1 [18]	X^1^			X^2^		
> 6 months after treatment	7 [20, 21, 23–26, 35]		X^5^			None apparent^6^	

^1^Only one study represented

^2^Small number of patients

^3^Large difference in populations hence diverging scores, despite same instrument used

^4^Only one study representing metastatic population, no studies representing neoadjuvant population

^5^Large range between scores despite comparable study populations, presumably due to different instruments used, incl. Non-validated instruments

^6^As estimated by funnel plot

### Ethical considerations

This study did not require national or institutional approval.

## Results

The search strategies in PubMed and Embase performed 2nd of July 2018, resulted in 103 and 105 scientific papers, respectively. All 208 titles were examined and if found relevant, abstracts and papers were read resulting in eight eligible papers from PubMed and eleven eligible paper from Embase. Four of these papers were overlapping. Thus fifteen papers were reviewed for the purpose of the study. Through cross-references a further six papers were included, resulting in a total of 21 eligible papers for review as illustrated in Fig. 1. The results are listed by topic in Table 1.

### Quality of life instruments

A total of 21 different QoL instruments were applied, most frequently the EORTC QLQ-C30, SF-36, FACT-G and FACT-BL, see Table 7, only displaying the validated instruments (*N* = 18). BC specific items were used in 52% of the included papers, FACT-BL being the most frequently used bladder specific measure. Five of the studies used non-validated questionnaires, some as a supplement to validated questionnaires, either developed by the investigator or modified from other validated questionnaires applied among other cancer groups, e.g., prostate cancer.

**Table 7:** List of quality of life instruments applied, *N* = 18

Abbreviated QoL instrument	Full Title
EORTC QLQ-C30	European Organization for Research and Treatment of Cancer Quality of life Core Questionnaire – for all cancer patients
EORTC QLQ-BLM30	European Organization for Research and Treatment of Cancer Quality of life module for muscle invasive bladder cancer
LENT-SOMA	Late Effects in Normal Tissue – Subjective, Objective, Management and Analytic scale for late effects of radiotherapy
SF-36	RAND Medical Outcomes Study Short Form 36
SF-12	RAND Medical Outcomes Study Short Form-12
EPIC	Expanded Prostate Cancer Index Composite
FACT-G	Functional Assessment of Cancer Therapy – General – for all cancer patients
FACT-BL	Functional Assessment of Cancer Therapy – for patients with bladder cancer
FACT-Taxane	Functional Assessment of Cancer Therapy – for patients receiving taxane therapy
IPSS	International Prostate Symptom Score
EuroQOL EQ-5D	EuroQOL Group non-disease specific QoL instrument
CTPS	Cancer Treatment Perception Scale
IOCv2	Impact of Cancer version 2
HADS	Hospital Anxiety and Depression Scale
WHOQOL-BREF	World Health Organization Quality of Life abbreviated version
STAI-10	State-Trait Anxiety Inventory-Trait scale short form
IIEF	International Index of Erectile Function
Spitzer index	Validated instrument for palliative patients

### Main topics

All but two of the included studies defined the focus areas as opposed to letting the patient define the areas of most concern. The main focus areas listed in order of declining frequency were global QoL (17/21, 81%), urinary symptoms (12/21, 57%), bowel symptoms (12/21, 57%), sexual function (10/21, 48%), fatigue (10/21, 48%), pain (8/21, 38%) and anxiety/depression (5/21, 24%). For the above listed focus areas, a graphical presentation by disease phase is presented in Fig. 2. Anxiety/depression was not included in Fig. 2 because of a limited number of studies with this focus. Other focus areas were financial distress, nausea, dyspnea, insomnia, appetite loss, rash and neuropathy, but all were only listed once.

The studies reporting global QoL all had subdivided QoL into the following health related quality of life domains: physical, mental, social, cognitive, emotional and personal function.

### Patients

Six of the 21 studies (29%) presented data from only MIBC patients, whereas eight studies showed data from both the NMIBC and MIBC populations. Four studies presented QoL scores for patients with metastatic or recurrent disease, two studies presented data for a mix of MIBC and metastatic patients and one study reported data on patients defined by the authors as ‘advanced’ BC patients receiving chemotherapy, thereby potentially including both patients undergoing neoadjuvant, curative intended chemotherapy for locally advanced BC and metastatic BC patients.

### Treatment

One study collected QoL data before cystoscopy. Thirteen of the 21 studies presented data from patients after receiving treatment, feasibly for comparison of two treatment modalities or to determine QoL for long-term survivors. Only two of these studies collected baseline data before treatment initiation. Seven studies measured QoL from patients undergoing chemotherapy, one of which was concurrent with radiotherapy in a Phase 1 trial, three presenting data from a Phase 2 trial and one from a Phase 3 trial. None of these studies had QoL as the primary outcome, and the Phase 1 trial was the only to include bladder cancer specific items. A total of six of the 21 studies did not list the QoL scores or were not on a comparable scale and were therefore not included in the analyses. The treatments consisted of combinations of transurethral resection, radical cystectomy, partial cystectomy,^1^ electrocoagulation, nephrouretherectomy, installation of Balcillus Calmette-Guérin and radiotherapy with or without concurrent chemotherapy. There was a large disparity between the studies as one study had mainly surgically treated patients in stages Ta-T1 and very few patients in need of adjuvant treatment for more advanced stages while another study presented patients volunteering for an inpatient rehabilitation after their oncological treatment suggesting more invasive treatment and sequelae thereof.

### Quality of life outcome

Two studies presented QoL before diagnosis as a baseline QoL not affected by the distress and change of perspective of having a cancer diagnosis [11, 12]. Goossens-Laan et al. showed significantly poorer QoL scores on erectile and orgasmic function in the BC group vs. the group with hematuria from other causes while Fung et al. displayed overall QoL data with a significant fall in Physical and Mental Component Scores (PCS, MCS) from pre- to post-diagnosis, although results were not clinically meaningful with small relative differences in the two groups. For MIBC patients this fall in PCS remained significant and clinically meaningful for all times after diagnosis and was greatest for patients with multiple comorbidities. This latter finding was echoed by Perlis et al. for patients post treatment [13].

When looking at QoL during treatment, the by far largest study by von der Maase et al. (reported by Roychowdhury et al.) with a total of 326 patients in two treatment arms, reported improvement in QoL during the chemotherapy treatment for metastatic patients. However, the results were not found significantly different from baseline values and no bladder specific items were used [14, 15]. The studies by Joly, De Santis, Herman and Albers all presented stable overall QoL scores during chemotherapy treatment, although in the Albers study this was, as for pain values, only seen for responders [16–19]. Likewise, the study by Joly et al. found an improvement in QoL among 10% of patients with objective response or stabilization of disease as a result of the treatment [16]. Herman et al. presented significantly lower bladder specific scores for patients receiving a higher dose of chemotherapy and lower overall QoL scores for those experiencing dose-limiting toxicities [18].

The QoL data from the after-treatment studies were collected from 0 to 37 years after BC diagnosis and treatment, rendering comparison somewhat difficult. However, for the patients having undergone radiotherapy as a bladder conserving strategy, the majority of the studies found the patients to have good or satisfactory bladder, bowel and/or sexual function and superior to that of cystectomy treated individuals when these were used as control groups [20–25]. Nonetheless, Fokdal et al. presented large impact on urinary, bowel and sexual function and reported high prevalence of disturbances; up to 94% impaired erective dysfunction while Lagrange et al. presented deteriorating over time. Comparison was in the Fokdal study made with population controls while Lagrange presented data from only 6–7 individuals at 36 months [26, 27].

The QoL scores from the studies above are displayed in Table 2 and gathered graphically in Fig. 2 displaying the overall QoL and subdivisions into urinary, bowel and sexual symptoms as well as pain and fatigue over the time course of a MIBC patient’s treatment. The GRADE evaluation was done for the overall QoL, urinary, sexual and bowel symptoms but not conducted for the outcomes fatigue and pain due to a very limited number of studies rendering GRADE analysis redundant.

Overall, we found that QoL has been immensely explored for MIBC patients post-treatment, free of disease, as shown by the GRADE analysis in Tables 3, 4, 5, 6. We found no studies reporting data during treatment for patients outside clinical trials, neither for the neoadjuvant nor metastatic population. From the summarized QoL scores in Fig. 2 it seems clear that especially urinary symptoms and sexual impairment are important issues for this group of patients. The GRADE analysis makes clear that Fig. 2 should be interpreted with caution due to the low level of evidence for almost all time-points.

## Discussion

To the best of our knowledge, this review is the first to compile quality of life studies in BC patients receiving medical oncological treatment. We have portrayed a diversity in choice of QoL questionnaires and an absence of studies informing us about QoL in the neoadjuvant and metastatic populations outside clinical trials. The summarized QoL curves in Fig. 2 lead us to believe that urinary symptoms and sexual impairment impacts QoL substantially due to their inverse relationship over the course of time. The developmental curve of overall QoL illustrates a tendency of increasing QoL after initiating treatment followed by a fall in the early months after treatment. Subsequently QoL increases in survivors more than 6 months after treatment. Also, only few studies included psychological items in the QoL instruments or as a supplement. However, a previous study showed that bladder cancer diagnosis did not significantly affect the patients’ levels of anxiety and depression [28], thus suggesting that QoL may not be significantly influenced by these issues in bladder cancer patients. Also, little attention has been paid to the psychosocial issues of the patients and the importance of these in relation to a person’s QoL, which ultimately could explain the reported levels of QoL in the different domains [29]. These issues are described in the literature in the general cancer population [30–33]. From these reports it is not evident in which direction psychosocial difficulties interfere with a patient’s QoL as the studies report diverging influences in the populations of interest, thereby rendering a need to understand how QoL is influenced by different psychosocial perspectives in the BC population.

Based on this review one may question whether there exists sufficient knowledge to reach the primary aim: to understand the QoL of BC patients undergoing medical oncological treatment. While the search string focused on patients in chemotherapy, radiotherapy studies were included because of the evident lack of post-treatment studies informing us about the QoL after chemotherapy treatment. The following apparent heterogeneity in content and design illustrated by the large variety of patients comprising either BC patients only with hematuria [12], recruited when in a clinical trial [14–16, 18, 19], recruited in post-treatment clinic [34] or applying methodologically problematic study designs challenging the implications of results such as a cross-sectional setting [11], determining QoL for patients having undergone radiotherapy without applying radiotherapy-specific questionnaires [20, 21, 23, 26, 34] or recruiting patients years after diagnosis with no baseline data [20, 21, 23–26, 35–38] renders careful conclusions about the development of QoL through a patient’s phases of disease.

Having addressed these issues, the most apparent outlier in Fig. 2 deserves notice. The unmistakable and permanent decrease in sexual impairment from before cystoscopy to before treatment may be the parameter to best distinguish MIBC patients in medical oncological treatment from MIBC patients undergoing surgical treatment. The latter are described as having a substantial amount of sexual troubles after cystectomy, because of the accompanying prostatectomy, a development not described in this review [39, 40]. Sexual problems are known to have a significant impact on QoL which makes this development in Fig. 2 a paramount finding in this review [41].

With the above issues in mind, this review is the first to gather the raw data from previous studies and create an overview of this diverse group of patients. Figure 2 may despite the apparent heterogeneity among the studies serve as the currently best guidance for physicians facing patients commencing medical oncological treatment. When discussing worries about QoL as a result of treatment, the results of this review may reassure patients unsure of future outcomes. Also, patients unsure of the effects of urological surgical interventions and searching for viable alternatives may need this platform of evidence to assist treatment decisions.

### Study limitations

Our attempt to align different QoL instruments and further align various time points applied in different populations studied and assuming an equal weight of each study presents a clear limitation of the present review. The GRADE system, although systematic, does not take into account the large cultural differences between two otherwise comparable populations; a Japanese patient may score QoL higher than an American patient despite objectively the same burden of symptoms, or vice versa. This may be the reason for the variation in scores as listed in Table 2 and commented in Tables 3, 4, 5, 6. Also, the combined scores of the FACT-BL instrument may both over- and underestimate the impairment in the urinary, bowel and sexual domains. The direction of this estimate cannot be determined. These issues constitute the need to interpret Fig. 2 with care, as illustrated by the GRADE analysis. Further, for some of the time points in this figure only one or two QoL scores make up the supposed overview of QoL development, perhaps falsely giving the single studies equal weight of time points comprising many studies. Unfortunately, this is a result of the scarce literature in this field and cannot be avoided. The scares literature in certain populations or time points may even give us valuable information of which patient groups have not been studied as intensively as others and guide us to future prospective research, in this case towards an understanding of the QoL for BC patients in neoadjuvant chemotherapy and for the metastatic population outside clinical trials. Lastly, although thorough literature search was performed in the two chosen databases including cross-references, the study group acknowledges that a number of studies describing QoL in advanced BC patients and specifically studies describing QoL during or after radiotherapy may, because of our chosen search string, not be included in our review. Also, given the nature of the QoL outcome and the psychological construct related to overall QoL, the results may have benefitted from a similar search in e.g. PsychInfo.

## Conclusions

As set out to do, this review sheds new light on the issues at hand for MIBC patients before, during and after medical oncological treatment. It provides a listing of QoL issues important for BC patients to include in prospective patient-reported outcome trials and identifies a need for further efforts to describe the QoL issues with validated instruments for advanced and metastatic BC patients, especially during treatment.

## Additional files

Additional file 1: PubMed search string. (PNG 44 kb)

Additional file 2: PRISMA Checklist. (DOC 63 kb)

## Footnotes

1 From Matsuda et al. [35] without further explanation. May be cystectomy in women without resection of internal genitalia.
