# Correction to: Quality of life in bladder cancer patients receiving medical oncological treatment; a systematic review of the literature

**DOI:** 10.1186/s12955-019-1247-1

**Published:** 2020-01-23

**Authors:** G. A. Taarnhøj, C. Johansen, H. Pappot

**Affiliations:** 1 Department of Oncology, Rigshospitalet, Blegdamsvej 9, section 5073, 2100 Copenhagen Ø, Denmark; 2 Unit of Survivorship, Danish Cancer Society, Strandboulevarden 49, 2100 Copenhagen Ø, Denmark

**Correction to: Health Qual Life Outcomes (2019**)**;17:20**

**https://doi.org/10.1186/s12955-018-1077-6**

The original article [1] contains a major error whereby a main Table is omitted. Thus, new Table 3 (shown ahead) should be considered as Table 3 in the original article. All citations in the text to tables are with these changes are correct.

**Table 3:** Outcome: Overall QoL

		GRADE issues					Overall GRADE rating
	Nb. of studies	Risk of bias	Inconsistency	Indirectness	Imprecision	Publication bias	
Before diagnosis	1 [12]	X^1^		X^2^	X^3^		
Before treatment	4 [14, 18-19, 27]		X^4^, X^5^				
During treatment	2 [16, 18]		X^4^	X^6^	X^3^		
≤ 6 months after treatment	4 [18-19, 27, 34]	X^7^	X^5^				
> 6 months after treatment	7 [13, 20-21, 24-25, 27, 35]	X^8^	X^9^, X^5^			None apparent^10^	

^1^ Two validated QoL questionnaires with very large difference between outcomes

^2^ Predominantly NMIBC patients, scores not specific for MIBC population

^3^ Small number of patients

^4^ Large difference in populations hence diverging scores

^5^ Use of different QoL scales

^6^ Only one study representing metastatic population, no studies representing neoadjuvant population

^7^ Mixed BC population, selected population [34]

^8^ Compliance 47-77%, thereby introducing selection bias

^9^ QoL questionnaires designed for prostate population

^10^ As estimated by funnel plot
