# Supplementary figures and images for: Quality of life in bladder cancer patients receiving medical oncological treatment; a systematic review of the literature
Source: Health Qual Life Outcomes. 2019 Jan 22;17:20. doi: 10.1186/s12955-018-1077-6 (PMC6341712; doi:10.1186/s12955-018-1077-6)

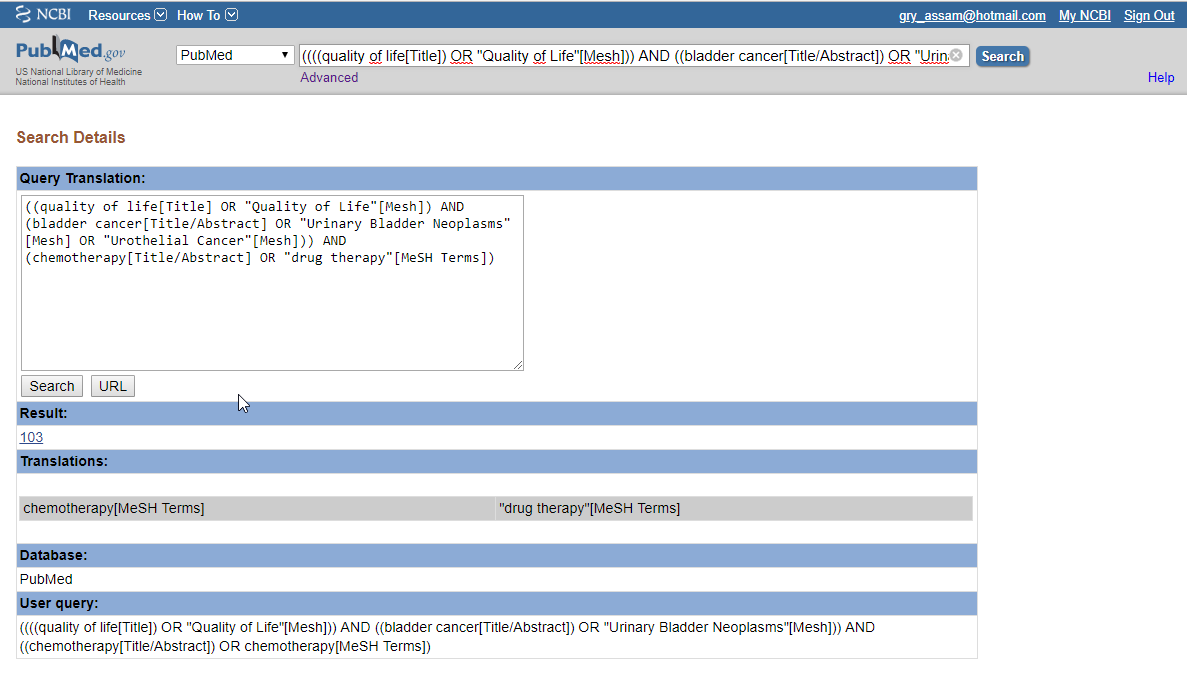

Supplement: Supplementary file 1 — PubMed search string. (PNG 44 kb) [file 12955_2018_1077_MOESM1_ESM.png]
